# Hidden in plain sight: Chlorhexidine as the culprit in recurrent perioperative anaphylaxis

**DOI:** 10.1016/j.jacig.2026.100763

**Published:** 2026-07-17

**Authors:** Josue A. Valdez, Emily Gansert, Victoria Hough, Alexei Gonzalez-Estrada

**Affiliations:** a Division of Allergy, Asthma and Clinical Immunology, Department of Medicine, Mayo Clinic, Scottsdale, Ariz; b Department of Internal Medicine, Mayo Clinic, Jacksonville, Fla; c Mayo Clinic Alix School of Medicine, Mayo Clinic, Scottsdale, Ariz

## Abstract

A 37-year-old renal transplant candidate experienced recurrent perioperative anaphylaxis during induction. Skin testing during a comprehensive allergy evaluation identified chlorhexidine allergy. After chlorhexidine avoidance and use of povidone iodine, the patient subsequently underwent successful renal transplantation without complications.

Perioperative anaphylaxis (POA) has an incidence of approximately 1 in 7000 procedures, with a mortality rate of 2% in the United States.^1^ Although neuromuscular blocking agents (NMBAs), antibiotics, and latex are the most reported triggers of POA, chlorhexidine (which is the most widely used antiseptic in surgical and procedural settings) has emerged as a hidden, yet increasingly recognized cause.^2^ Recent data also suggest that beyond its use in skin preparation and catheter coatings, chlorhexidine-containing devices, such as central venous or pulmonary artery catheters, may contribute to perioperative reactions that are often misattributed to more common agents, leading to delays in diagnosis and management.^3^ This highlights the importance of including chlorhexidine in the differential diagnosis when evaluating POA. The patient underwent informed consent.

A 37-year-old male with end-stage renal disease (ESRD) due to IgA nephropathy and no history of drug allergy was scheduled for renal transplantation. During transplant induction of anesthesia for the transplant procedure at an outside hospital (OSH), the patient experienced 2 separate anaphylactic episodes, leading to procedure cancellations. The first episode occurred after induction with propofol, fentanyl, and rocuronium and after the patient had received cefazolin and lidocaine, causing tachycardia, hypotension (a systolic blood pressure of 53 mm Hg), and elevated positive end-expiratory pressure (12 cm of H_2_O), requiring norepinephrine and 2 doses (1 mg in total) of intravenous epinephrine via drip. The patient was intubated and transferred to the intensive care unit, with improvement of his symptoms. The OSH documentation of medication dosing for this event was incomplete.

Nine months later, a second attempt at anesthesia induction using vecuronium, 10 mg, and etomidate, 15 mg, instead of rocuronium and propofol led to a similar reaction (a systolic blood pressure of 40 mm Hg). The patient also received fentanyl (250 μg), cefazolin (2 g), methylprednisolone (500 mg), and lidocaine (20 mg) before the reaction. He required a phenylephrine drip (0.2 mg), norepinephrine drip (1.82 mg), vasopressin drip (23.2 units), epinephrine push and drip (2.38 mg), methylene blue (10 mg), and sugammadex (200 mg), as well as care in the intensive care unit care. No acute serum tryptase levels were obtained during either event.

The patient then underwent a comprehensive allergy evaluation at our institution. The laboratory results showed a baseline serum tryptase level of 17.2 ng/mL (reference level < 11.5 ng/mL), which was likely attributable to ESRD.

Skin testing to all possible causal agents used before the POA episodes (cefazolin, major and minor penicillin determinants, rocuronium, vecuronium, etomidate, propofol, fentanyl, lidocaine, and methylprednisolone) was performed 14 months after the last POA episode (Table I). Our current practice includes skin testing to causal agents (ie, antiseptics [chlorhexidine and povidone iodine] and latex) that are commonly not documented in anesthetic records (Table I). We also include potential alternative NMBAs available at our institution (Table I) in the event of identification of an NMBA during skin testing. Skin prick testing with chlorhexidine yielded a positive result (10.5-mm wheal and 15.5-mm flare). The results of testing (both skin prick and intradermal testing) for all of the other tested agents were negative. Negative and positive controls we also included during the skin testing. The level of latex serum-specific IgE was less than 0.10 kU/L (reference level < 0.70 kU/L), and the patient tolerated graded challenges to oral trimethoprim-sulfamethoxazole, subcutaneous lidocaine, and cutaneous and inhaled exposure to latex. Trimethoprim-sulfamethoxazole challenge was requested by the anesthesia team because of the central venous catheters containing sulfadiazine. Latex challenge is performed by placing a powdered latex glove on 1 hand for 10 minutes, followed by 15 minutes of observation. If the result is negative, the patient then inhales air released from a powdered latex glove held in front of the face and is observed for an additional 15 minutes.^4^

**Table 1:** Perioperative anaphylaxis skin testing protocol

Causal agent	Stock concentration	SPT result (mg/mL)		IDT result (mg/mL)	
		Dilution	Maximal concentration	Dilution	Maximal concentration
Antibiotics					
Benzylpenicilloyl polylysine (M)	6.0 x 10^–5^	Undiluted	6.0 × 10^–5^	Undiluted	6.0 × 10^–5^
Cefazolin (mg/ML)	330	Undiluted	330	1:10	33
Penicillin G (U/mL)	10,000,000	1:1,000	10,000	1:1,000	10,000
NMBAs (mg/mL)∗					
Atracurium	10	Undiluted	10	1:1,000	0.01
Cisatracurium	2	Undiluted	2	1:100	0.02
Rocuronium	10	Undiluted	10	1:200	0.05
Succinylcholine	20	Undiluted	20	1:25	0.8
Vecuronium	10	Undiluted	1	1:100	0.01
NMBA reversal agents (mg/mL)∗					
Neostigmine	1	Undiluted	1	1:5	0.2
Sugammadex	100	Undiluted	100	1:2	50
Sugammadex-rocuronium complex (mg/mL)∗	35.7 + 10	Undiluted	35.7 + 10	1:5	7.1 + 2
Antiseptics(mg/mL)					
Chlorhexidine gluconate 4%†	40	1:8	5	1:20,000	0.002
Povidone iodine	100	Undiluted	100	NA	NA
Latex patch with normal saline	NA	Undiluted	NA	NA	NA
Hypnotics (mg/mL)					
Dexmedetomidine	0.004	Undiluted	0.004	1:1,000	0.000004
Diazepam	5	Undiluted	5	1:1,000	0.005
Etomidate	2	Undiluted	2	1:10	0.2
Ketamine	10	Undiluted	10	1:10	1
Midazolam	5	Undiluted	5	1:50	0.1
Opioids (mg/mL)					
Alfentanil	0.5	Undiluted	0.5	1:10	0.05
Fentanyl	0.05	Undiluted	0.05	1:10	0.005
Morphine	10	1:10	1	1:1,000	0.01
Hydromorphone	2	Undiluted	2	1:1,000	0.002
Remifentanil	0.05	Undiluted	0.05	1:10	0.005
Sufentanil	0.05	Undiluted	0.05	1:1,000	0.00005
Blue dyes (mg/mL)					
Isosulfan blue/sulfa blue 1%	10	Undiluted	10	1:10	1
Methylene blue	10	Undiluted	10	1:100	0.1
Patent blue	25	Undiluted	25	1:100	0.25
Colloids (mg/mL)					
Gelatin	560	Undiluted	560	NA	NA
Corticosteroids, including the following excipients (mg/mL)					
Carboxymethylcellulose 1%	10	Undiluted	10	1:10	1
Dexamethasone	4	Undiluted	4	1:10	0.4
Hydrocortisone	50	Undiluted	50	1:100	0.5
Methylprednisolone acetate	40	Undiluted	40	1:10	4
Methylprednisolone succinate	40	Undiluted	40	1:10	4
Polyethylene glycol 3350	17	Undiluted	17	NA	NA
Local anesthetics (mg/mL)					
Bupivacaine 0.25%	2.5	Undiluted	2.5	1:100	0.025
Chloroprocaine 1%	10	Undiluted	10	1:100	0.1
Lidocaine 1%	10	Undiluted	10	1:100	0.1
Ropivacaine 0.5%	5	1:2.5	2	1:10	0.5
Tetracaine 1%	10	Undiluted	10	1:100	0.1

*IDT*, Intradermal testing; *NA*, nonapplicable; *SPT*, skin prick testing.

∗ See Table I in Gonzalez-Estrada et al for the sugammadex-rocuronium complex preparation. J Allergy Clin Immunol Pract 2023;11:466-73.

† Diluted with sterile water for SPT and with normal saline for IDT per Shah et al. J Allergy Clin Immunol Glob 2024;4:100372.

The patient and the anesthesia team at the OSH were instructed to avoid chlorhexidine and use alternative antiseptics such as povidone iodine for future procedures. The patient subsequently underwent a successful transplantation procedure, with use of povidone iodine as an antiseptic and no further reactions.

In many cases, the culprit agent remains unidentified, with up to 40% of reactions lacking a confirmed diagnosis despite comprehensive testing.^3^ This diagnostic uncertainty complicates perioperative care. POA occurs in approximately 1 in 7000 procedures in the United States and is more common in transplant operations.^1^ Ideally, skin testing should be performed within 4 to 6 weeks after the index reaction.^5^ Early skin testing (at <4 to 6 weeks) after POA may be considered when urgent reoperation is required; however, it is associated with lower culprit identification rates and potential false-negative results, often requiring repeat testing after 4 to 6 weeks. In contrast, late testing beyond 6 weeks does not appear to reduce diagnostic yield significantly.^6^^,^^7^

Our patient experienced 2 reproducible life-threatening reactions; a clear culprit, namely, chlorhexidine, which is a well-recognized perioperative allergen, was identified through skin testing.^3^ This patient emphasizes the importance of a through allergy evaluation after the first occurrence of POA. Recent multicenter data suggest that recurrence after comprehensive perioperative allergy evaluation is uncommon.^8^ Chlorhexidine testing using a recently described protocol with sterile water for skin prick testing and normal saline for intradermal dilutions was performed to avoid precipitation and false-negative results.^9^ There are commercially available serum-specific IgE preparations to chlorhexidine in Europe, but they are not available in the United States. Although sensitization to alpha-gal remains a potential concern, our current practice is to pursue alpha-gal testing primarily in patients with known exposure to potential cross-reactive agents such as heparin or when an initial comprehensive evaluation fails to identify a causal agent.

Interpretation of baseline serum tryptase level was complicated by the patient’s ESRD, which can elevate baseline values. Limitations of this case report include the fact that acute serum tryptase samples were not collected during either POA episode, the OSH records lacked doses for medications for the first POA episode, and we do not currently perform anesthetic challenges in our practice. The current guidelines recommend measuring both acute and baseline tryptase levels to confirm mast cell activation and improve diagnostic accuracy in POA.^3^^,^^9^

Accurate identification of chlorhexidine sensitization has critical clinical implications, enabling targeted avoidance in future procedures and preventing nonspecific allergy labels. Because perioperative chlorhexidine exposure may be concealed in catheters and other devices, this case highlights the value of a comprehensive allergy evaluation to guide patient safety and management.

## Disclosure statement

Disclosure of potential conflict of interest: The authors declare that they have no relevant conflicts of interest.
